# Study on Performance Improvements in Perovskite-Based Ultraviolet Sensors Prepared Using Toluene Antisolvent and CH_3_NH_3_Cl

**DOI:** 10.3390/nano11041000

**Published:** 2021-04-13

**Authors:** Seong Gwan Shin, Chung Wung Bark, Hyung Wook Choi

**Affiliations:** Department of Electrical Engineering, Gachon University, 1342 Seongnam Daero, Seongnam-Si 13120, Korea; 1020days@gmail.com (S.G.S.); bark@gachon.ac.kr (C.W.B.)

**Keywords:** ultraviolet (UV) sensors, bandgap widening, CH_3_NH_3_PbBr_3_, antisolvent, CH_3_NH_3_Cl

## Abstract

In this study, a simply structured perovskite-based ultraviolet C (UVC) sensor was prepared using a one-step, low-temperature solution-processing coating method. The UVC sensor utilized CH_3_NH_3_PbBr_3_ perovskite as the light-absorbing layer. To improve the characteristics of CH_3_NH_3_PbBr_3_, an antisolvent process using toluene and the addition of CH_3_NH_3_Cl were introduced. The device with these modifications exhibited a response rise/fall time of 15.8/16.2 ms, mobility of 158.7 cm^2^/V·s, responsivity of 4.57 mA/W, detectivity of 1.02 × 10^13^ Jones, and external quantum efficiency of 22.32% under the 254-nm UV illumination. Therefore, this methodology could be a good approach in facilitating UVC detection.

## 1. Introduction

Ultraviolet (UV) sensors have received significant research interest owing to their promising applications in communications, image sensing, environmental monitoring, astronomy, and medicine [1,2,3,4,5]. The UV range can be divided into three areas depending on the wavelength range, of UVA (320–400 nm), UVB (280–320 nm), and UVC (100–280 nm). Of the UV rays emitted by the sun, most UVA radiation reaches the Earth’s surface, most UVB is absorbed by the ozone layer with only a small amount reaching the surface, and UVC is completely absorbed by the ozone layer and atmosphere and does not reach the Earth surface.

While UVC is rare on the surface of the Earth, it can be emitted from lightning strikes or artificial sources such as arc welding, mercury lamps, and UV sterilization lamps. Even extremely low exposure to UVC can have a detrimental effect on the human body. The short-wavelength UV rays of less than 300 nm do not penetrate the epidermis when they touch the skin. However, long-wavelength UV rays have a strong penetrating power and can adversely affect the skin, eyes, and immune system, and are considered as harmful to the human body. Because UV rays are invisible to human vision, many safety accidents are caused by exposure to UV rays at industrial sites.

In addition, when the insulation levels of high-voltage transmission lines and transformers have deteriorated, power loss can occur via arc discharge. With continuous discharge, the insulating facility deteriorates further and the power system is destroyed; thus, large-scale power failure may occur.

Therefore, it is essential to develop semiconductor-based optical sensor systems that can convert incident radiation signals into electrical signals. In general, semiconductor-based sensors employ two detection methods. The first method uses a semiconductor with a wide bandgap, such as AlGaN, MgZnO, Ga_2_O_3_, ZnGa_2_O_4_, MoS_2_, or diamond. However, these materials require high-temperature and expensive processing methods, such as molecular beam epitaxy, chemical vapor deposition, pulsed laser deposition, atomic layer deposition, and magnetron sputtering [6,7,8,9,10]. The second detection method uses a narrow-bandgap Si diode sensor equipped with a UV filter. However, the high-performance UVC detection is difficult to achieve with Si diodes, because photogenerated carriers cannot easily reach the depletion layer of the semiconductor.

Recently, organic–inorganic perovskites have received much attention in radiation emission and detection, including solar cell applications. The high-quality perovskite crystals can be grown easily using a simple cold-solution method. The perovskite thin films coated by a simple low-temperature solution method can be uniform with good pinhole control by increasing the nucleus density of the perovskite through an antisolvent process. In addition, when Br^−^ ions are substituted with Cl^−^, the bandgap increases. Perovskites have a large absorption coefficient of approximately 10^5^ cm^−1^ in the UVC spectral range, as well as high mobility. Therefore, perovskite materials are expected to show high sensitivity and fast UVC response performance in UVC detection sensors when used with UV filters. However, the use of perovskites for UVC detection has rarely been reported. With the addition of an antisolvent process, a uniform and pinhole-controlled film can be manufactured, and by adding CH_3_NH_3_Cl, the bandgap is adjusted, resulting in improved UVC performance.

In this study, a CH_3_NH_3_PbBr_3_ thin film was fabricated on an etched indium-doped tin oxide (ITO) electrode using a one-step process to form a perovskite film-based UV sensor. To improve the surface of the prepared CH_3_NH_3_PbBr_3_ thin film and control the bandgap, a UVC sensor with a simple structure was fabricated through an antisolvent process with the addition of CH_3_NH_3_Cl. Consequently, the modified CH_3_NH_3_PbBr_3_ thin film exhibited improved surface properties, bandgap control, and mobility compared to those of the bare CH_3_NH_3_PbBr_3_. In addition, the perovskite-based UVC sensor exhibited a responsiveness of 4.57 mA/W, detectability of 1.02 × 10^13^ Jones, and an external quantum efficiency (EQE) of 22.32% under the 254-nm UV illumination. Besides, the simply structured UVC sensor fabricated using a low-temperature solution process showed high detectivity. The reported perovskite-based UVC sensors yield improved UVC sensing using materials with potential for further development.

## 2. Materials and Methods

### 2.1. Materials

Lead(II) bromide (PbBr_2_, 99.999% trace metals basis), N,N-dimethylformamide (DMF, 99.8%), toluene (99.8%), and dimethyl sulfoxide (DMSO, 99.7%) were purchased from Sigma Aldrich (Saint Louis, MO, USA). Methylammonium bromide (CH_3_NH_3_Br, MABr) and methylammonium chloride (CH_3_NH_3_Cl, MACl) were obtained from GreatCell Solar (Queanbeyan, Australia). ITO deposited on a quartz glass substrate with a thickness of 150 nm was obtained from RND Korea (Gwangmyeong, Korea). AZ GXR-601 photoresist (PR) and AZ 300 MIF were obtained from AZ Electronic Materials (Wiesbaden Germany). All materials were used without further purification.

### 2.2. Preparation of ITO

The quartz glass on which ITO was deposited was washed with distilled water, acetone, and 2-propanol for 20 min each with an ultrasonic cleaner. The washed ITO glass was then dried in nitrogen and 50 μL of AZ GXR-601 solution was spin-coated onto the dried substrate at 4000 rpm for 60 s. The coated PR solution was fired at 95 °C for 1 min and then cooled to room temperature. Subsequently, UV irradiation was performed for 100 s in an exposure machine using a patterned photomask. After the exposure process was completed, the ITO glass was immersed in AZ 300 MIF to remove the part exposed to UV, and then fired at 120 °C for 1 min and cooled to room temperature. The ITO was etched using an ITO etching solution and washed with acetone and distilled water to remove the AZ GXR-601 residue.

### 2.3. Synthesis of Perovskite

The MAPbBr_3_ solution was mixed using a 1:1 molar ratio of PbBr_2_ to MABr, and 100 μL of DMSO was added and mixed in 1 mL of DMF. The mixture was stirred at room temperature for 6 h. After stirring, the solution was filtered through a 0.45-μm syringe filter just before coating. In the solution to which MACl was added, PbBr_2_, MABr, and MACl were dissolved in 1 mL of DMF at molar ratios of 1:1:0.05–0.20 (in 0.05-M increments), respectively, and 100 μL of DMSO was added. The resulting solution was stirred at room temperature for 6 h. The stirred solution was filtered through a 0.45-μm syringe filter just before coating and use.

### 2.4. Preparation of a UV Sensor Based on Perovskite Films

**Figure 1:**
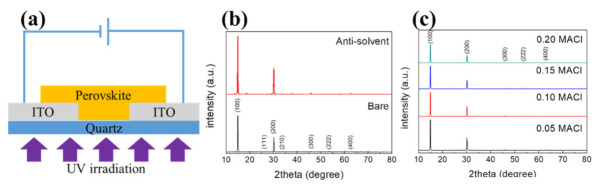
(**a**) Schematic of the fabricated sample and XRD patterns of MAPbBr_3_: (**b**) bare and antisolvent-processed, (**c**) with 0.05, 0.10, 0.15, and 0.20 M added MACl.

To fabricate the perovskite sensor, an etched quartz ITO substrate was first washed in distilled water, acetone, and 2-propanol with ultrasonic waves for 20 min each. After removing the residue by blowing nitrogen on the washed substrate, it was dried for 30 min at 80 °C in a dryer. Polyimide tapes were attached to both ends of the dried ITO substrate to expose the electrodes. To prepare the MAPbBr_3_ thin film, 50 μL of the MAPbBr_3_ solution was dropped onto the etched ITO substrate and spin-coated at 3000 rpm for 45 s. In order to introduce an antisolvent process, MAPbBr_3_ was coated by dropping 60 μL of toluene for 25 s during coating. The coated MAPbBr_3_ thin film was fired at 140 °C for 15 min and then cooled to room temperature. For comparison, a bare sample without the antisolvent processing was also produced. A schematic of the fabricated device is shown in Figure 1a.

### 2.5. Characterization and Device Measurement

The shape and microstructure of the synthesized perovskite layer were investigated by field-emission scanning electron microscopy (FE-SEM, S-4700, Hitachi, Tokyo, Japan) and X-ray diffractometry (XRD, D/MAX-2200, Rigaku, Tokyo, Japan) at the Smart Materials Research Center for IoT of Gachon University. The electrical properties of the devices were measured using a UV–visible (UV-vis) spectrometer (UV-Vis 8453, Agilent, Santa Clara, CA, USA). The electrical properties of the devices were examined using a semiconducting characterization system (2400 Sourcemeter, Keithley, Cleveland, OH, USA) equipped with a probe station (M 150, Cascade, Beaverton, OR, USA). A 254-nm UV lamp (VL6.LC, Vilber, France) was used as the light source for UV irradiation.

## 3. Results and Discussion

### Characteristics of the Prepared Perovskite Film

The XRD patterns of the bare MAPbBr_3_ and MAPbBr_3_ with the antisolvent process are shown in Figure 1b. The pattern of MAPbBr_3_ showed sharp diffraction peaks at 2θ = 14.94°, 26.02°, 30.16°, 33.92°, 45.90°, 53.56°, and 62.66°, corresponding to the (100), (111), (200), (210), (300), (222), and (400) planes, respectively [11,12,13]. The prepared MAPbBr_3_ thin films show no impurity diffraction peaks and the uniform pure tetragonal phase coatings are formed. The crystallite sizes, calculated using the full widths at half maximum and the Debye–Scherrer equation, are approximately 41.9 and 38.1 nm for the bare and antisolvent-processed samples.

The XRD patterns of the MAPbBr_3_ thin films with 0.05, 0.10, 0.15, and 0.20 M of added MACl are given in Figure 1c. The sharp peaks corresponding to the (100), (200), and (300) planes in each XRD pattern confirm that the MAPbBr_3_ thin films with 0.05–0.20 M added MACl were all in pure phase state. The peaks shifted to larger diffraction angles as the Cl content increased. The XRD peak of the (100) plane of the MAPbBr3 thin film to which 0.20 M of MACl was added was shifted from 14.94° to 14.96°. The measured d_100_ spacing of the MAPbBr_3_ thin film decreased from 2.995 Å (at 14.94°) to 2.983 Å (at 14.96°) with the addition of MACl, owing to the mixing of Cl with a smaller atomic radius [14,15,16].

**Figure 2:**
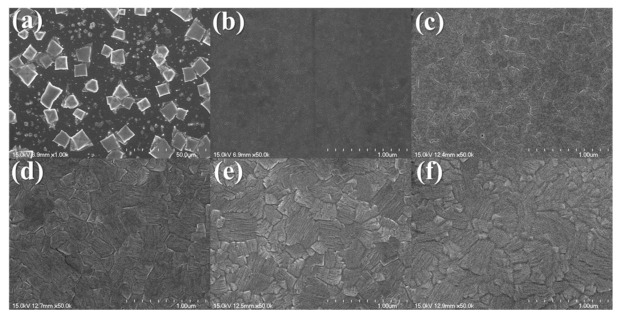
FE-SEM images of MAPbBr_3_: (**a**) bare, (**b**) antisolvent-processed, and with (**c**) 0.05 M, (**d**) 0.10 M, (**e**) 0.15 M, (**f**) 0.20 M added MACl.

The SEM images of the MAPbBr_3_ thin film are presented in Figure 2. The cubic shape MAPbBr_3_ crystal are seen in Figure 2a. The size of the crystals ranged from 5 to 15 µm. The crystal grains were separated, and because of the one-step coating method, in most cases, the surface was not wholly covered, and the crystal distribution was not uniform [17,18]. In Figure 2b, an SEM image is shown for the MAPbBr_3_ thin film with the antisolvent process. Compared with Figure 2a, the antisolvent-processed MAPbBr_3_ thin film showed a smooth and pinhole-controlled perovskite surface. The toluene-added MAPbBr_3_ thin films exhibited superior crystallinity and uniformity compared to the films attained through the conventional spin-coating method. It is known that the uniformity of the perovskite thin film can significantly affect the UV sensor performance [19,20].

In Figure 2c–f, the surfaces of the MAPbBr_3_ thin film with 0.05–0.20 M MACl are shown. The surfaces of the MAPbBr_3_ thin films to which 0.05–0.20M of MACl are added are thin films with large grain sizes. In Figure 2a–c, only relatively small grains were observed. In Figure 2d,e, the larger grains are observed due to grain growth, while Figure 2f shows blurry grain boundaries due to degradation from the intense chemical treatment. As the amount of MACl was increased, the grain size generally increased, while, in the MAPbBr_3_ thin film containing 0.10 M MACl, the grains of approximately 100 nm remained. However, large grains of 500 nm or more were dominant.

**Figure 3:**
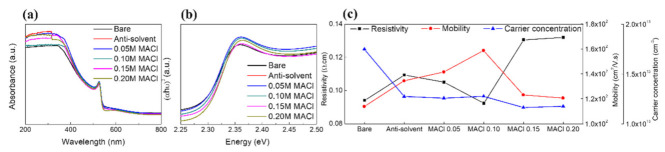
(**a**) UV-vis absorbance of MAPbBr_3_, (**b**) dependence of absorption of the photon energy, (**c**) electrical characteristics of MAPbBr_3._

The UV-vis absorbance spectrum of the MAPbBr_3_ thin film is shown in Figure 3a. MAPbBr_3_ has a strong absorption band in the range of 200–510 nm [21,22]. Therefore, a UV sensor based on MAPbBr_3_ has a strong advantage in detecting UV rays in the UVC region. The sharp band edge of MAPbBr_3_ was clearly observed, indicating the direct bandgap of MAPbBr_3_. The bandgap of the bare MAPbBr_3_ was estimated by extrapolating the linear range from (F(R∞)hν)^2^ to the photon energy (hν) intercept, depending on estimations by the Tauc and Davis–Mott models [23,24], is 2.27 eV [25,26]. The bandgap of the antisolvent-processed MAPbBr_3_ was 2.28 eV; the crystal size and the bandgap of the film decreased and increased, respectively, compared to those of the bare film. This is because the electron–hole pairs were much closer with non-negligible Coulomb interactions, resulting in a higher overall kinetic energy [27]. The bandgaps of MAPbBr_3_ with 0.05–0.20 M added MACl increased as the concentration of MACl increased.

The electrical characteristics of the MAPbBr_3_ thin films are shown in Figure 3c. The electrical characteristics, including the semiconductor type, resistivity, carrier concentration and mobility were determined using the Hall measurements. From the measurements, it was confirmed that all MAPbBr_3_ thin films had carrier concentrations of more than 10^13^ cm^−3^ and p-type characteristics. The resistivity values were 0.0941 and 0.1094 Ω·cm for the bare and antisolvent-processed MAPbBr_3_ thin films, and those for the MAPbBr_3_ films with 0.05, 0.10, 0.15, and 0.20 M MACl were 0.1051, 0.0924, 0.1306, and 0.1320 Ω·cm, respectively. The mobilities were 113.8, 134.4, 141.5, 158.7, 123.1 and 120.6 cm^2^/V·s for bare MAPbBr_3_, antisolvent-processed MAPbBr_3_, and MAPbBr_3_ with 0.05, 0.10, 0.15, and 0.20-M added MACl, respectively. The mobility increased as the amount of MACl was increased to 0.10 M, while it decreased for amounts exceeding 0.10 M. This was attributed to the increase in the diffusion distance and the decrease in the trap density with the addition of MACl. [28] The diffusion coefficient can be specified using the Einstein equation ($D=\frac{\mu K_{B}T}{q}$, where *K_B_* is the Boltzmann constant, *T* is the temperature of the sample, and *q* is the amount of charge). Because the diffusion coefficient is proportional to the diffusion distance, a higher mobility can lead to an increased diffusion distance.

**Figure 4:**
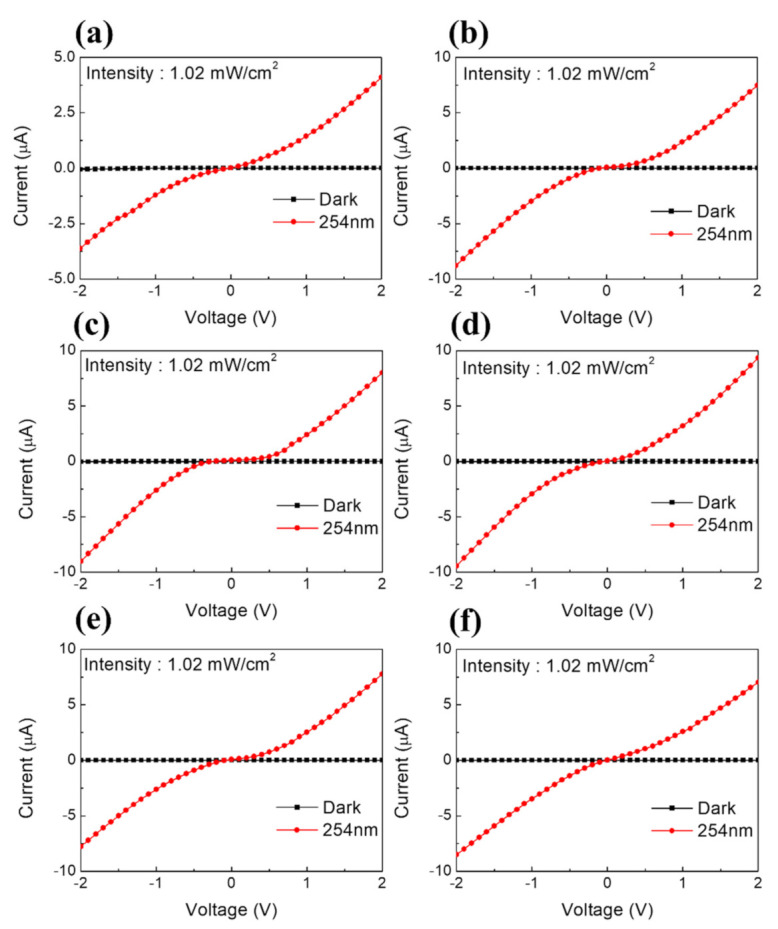
Current-voltage (*I–V*) characteristics of perovskite-based UV sensors. (**a**) Bare, (**b**) antisolvent-processed, and containing (**c**) 0.05-M MACl, (**d**) 0.10-M MACl, (**e**) 0.15-M MACl, (**f**) 0.20-M MACl.

To analyze the UVC detection characteristics, a series of sensor structures were fabricated and compared using bare MAPbBr_3_, antisolvent-processed MAPbBr_3_, and MAPbBr_3_ with 0.05–0.20 M MACl thin films deposited on the etched ITO. The current–voltage (*I–V*) curves of the MAPbBr_3_-based sensors are displayed in Figure 4. The *I–V* characteristics were measured by switching the bias voltage from −2 V to 2 V at a scan rate of 0.1 V in a dark room where light was blocked and under a 254-nm light source with 1.02 mW/cm^2^ output.

The coated perovskite formed a Schottky barrier owing to contact with the ITO electrode. Under the applied voltage, ion movement and carrier trapping in the active region occurred at the ITO/perovskite interface. This indicated that an ohmic contact was formed between the perovskite and ITO electrodes. For all samples, an apparent increase in current was detected under the 254 nm light at 1.02 mW/cm^2^. The *I–V* curves obtained under the 254 nm irradiation maintained rectified shapes even with the addition of various concentrations of MACl. In this measurement, the MAPbBr_3_ films with the addition of MACl showed that a significant photocurrent could be photogenerated.

The reactivity (*R*) and specific detectivity (D*) are the main parameters used to evaluate the UV sensors. *R*, which reflects the sensor response to incident light, is determined as *R* = (*I*_light_ − *I*_dark_)/*AP*_op_, where *I*_light_ is the photocurrent under 254-nm UV light, *I*_dark_ is the dark current, A is the active area of the sensor and *P*_op_ is the incident light power [29,30]. The detectivity D* reflects the performance of the sensor, which can be checked by the performance of the signal generated by the main noise and light source in the dark [31,32]. D* is expressed as D* = *R*/(2*qJ*_dark_), where *q* is the amount of charge and *J*_dark_ is the dark current density.

**Figure 5:**
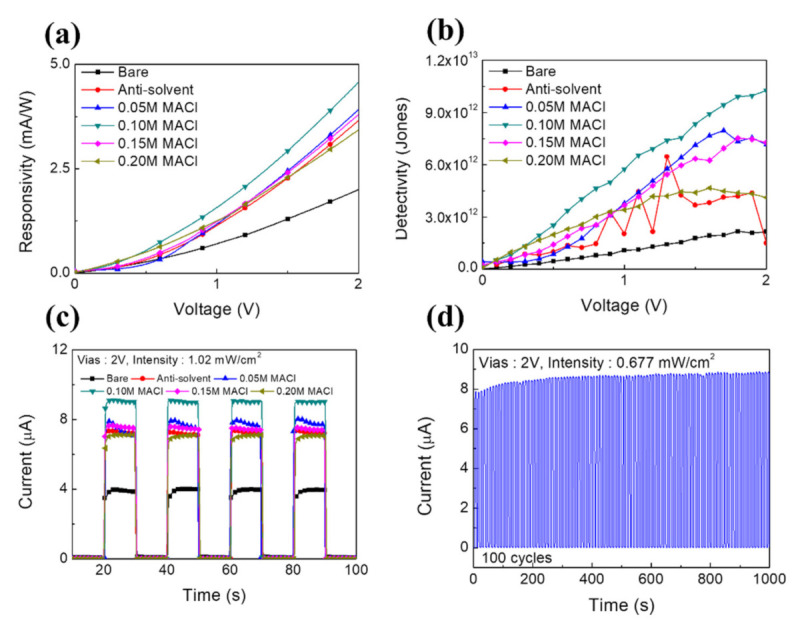
(**a**) Responsivity curves of MAPbBr_3_ sensors, (**b**) detectivity curves of MAPbBr_3_ sensors, (**c**) transient photoresponses, (**d**) stability of the prepared device for 100 ON/OFF switching cycles under the illumination of 254-nm light with an intensity of 0.677 mW/cm^2^.

In Figure 5a, the responsivity curves of the MAPbBr_3_-based sensors are given. The responsivity curves showed that the value of *R* gradually increased as the bias voltage increased from 0 to 2 V. This was attributed to the increase in the conversion efficiency of the photodetector from photons to charge. At the bias voltage of 2 V under the 254-nm illumination at 1.02 mW/cm^2^, the *R* values were 2.00, 3.65, 3.91, 4.57, 3.79 and 3.43 mA/W for bare, antisolvent-processed, 0.05-, 0.10-, 0.15- and 0.20-M MACl-added MAPbBr_3_, respectively.

The detectivity curves of MAPbBr_3_ with added MACl are shown in Figure 5b. Under an applied 2V bias voltage, the D* values were 2.13 × 10^12^ and 1.49 × 10^12^ for bare and antisolvent-processed MAPbBr_3_. The D* values were 7.14 × 10^12^, 1.02 × 10^13^, 7.28 × 10^12^, and 4.11 × 10^12^ for MAPbBr_3_ with 0.05, 0.10, 0.15, and 0.20 M of added MACl. Because D* depends on the dark current value, it is strongly influenced by the noise generated in measuring the dark current.

The EQE, another determinant of sensor performance, is defined as the number of electrons generated per incident photon [33,34,35], as follows: EQE = *Rhc*/*e*λ, where *h* is Planck’s constant, *c* is the speed of light, and λ is the wavelength of the incident light. At 2 V, EQEs were 9.77% and 17.84% for the bare and antisolvent-processed MAPbBr_3_, and 19.13%, 22.32%, 18.55% and 16.76% for MAPbBr_3_ with 0.05, 0.10, 0.15 and 0.20 M of added MACl, respectively.

In Figure 5c, the time-dependent photoresponses are shown for MAPbBr_3_-based sensors measured at a bias voltage of 2V and power intensity of 1.02 mW/cm^2^. The response time reflects the capacity of the photodetector to follow rapidly changing optical signals. The rise and fall times characterize the response speed of a light detector, and they are usually defined as the time intervals required for the photocurrent to rise, or fall, from 10 to 90% of its peak value, or vice versa. All fabricated samples showed excellent response characteristics, with rise/fall times of 32.6 ms/32.1 ms and 30 ms/28 ms for bare and antisolvent-processed MAPbBr_3_ and 29.8 ms/29 ms, 15.8 ms/16.2 ms, 25.8 ms/32.1 ms, and 35.1 ms/39 ms for MAPbBr_3_ with 0.05, 0.10, 0.15, and 0.20-M added MACl, respectively.

The ON/OFF repeatability of the operation of a sensor using an MAPbBr_3_ thin film with 0.10 M of added MACl under a 254-nm light source with 0.677 mW/cm^2^ output is given in Figure 5d. The sensor was irradiated for 5 s. The tested sensor showed reproducibility with consistent performance over 100 ON/OFF iterations. In the first iteration, the photocurrent was 7.06 μA; and after 100 repetitions was 8.78 μA. This shows that the reproducibility was maintained continuously relative to the initial photocurrent.

The parameters of the simply structured perovskite-based UVC detector developed in this study are similar to those of the photodetectors reported in other studies (Table 1). Compared to previously reported photodetectors, the proposed detector can be manufactured with a simple structure using a solution process at a low temperature, and it shows high-performance detection.

## 4. Conclusions

In summary, a simply structured perovskite-based optical sensor was fabricated through a low-temperature solution process. It showed a fast response speed (rise/fall time) of 15.8/16.2 ms, mobility of 158.7 cm^2^/V·s, responsivity of 4.57 mA/W, detectivity of 1.02 × 10^13^ Jones, and EQE of 22.32%. Therefore, the sensor reported in this study provides a promising solution for UVC detection.

## Figures and Tables

**Table 1:** Comparison of important parameters of various UV detectors.

Materials	Light (nm)	Method	Voltage (V)	Responsivity (mA/W)	Detectivity (Jones)	EQE (%)
CH_3_NH_3_PbBr_3_ [this study]	254	Solution	2	4.57	1.02 × 10^13^	22.2
CH_3_NH_3_PbCl_3_ [36]	255	Single crystals	5	450	-	219
CH_3_NH_3_PbBr_3_ [36]	255	Single crystals	5	300	-	146
CH_3_NH_3_PbI_3_ [36]	255	Single crystals	5	120	-	58
CH_3_NH_3_PbCl_3_ [37]	365	Single crystals	15	46.90	1.2 × 10^10^	-
Ga_2_O_3_ [38]	185	MOCVD *	10	0.3	2.8 × 10^10^	0.2
CsPbBr_3_-Cs_4_PbBr_6_ [39]	254	Vapor	0	49.40	1.2 × 10^12^	31
CH_3_NH_3_PbCl_3_ [40]	398	Solution	−1	71	1.2 × 10^10^	23

* Metal–organic chemical vapor deposition.

## Data Availability

Not applicable.
